# High prevalence of dyslipidaemia among persons with diabetes mellitus and hypertension at a tertiary hospital in Blantyre, Malawi

**DOI:** 10.1186/s12872-022-03011-y

**Published:** 2022-12-22

**Authors:** Kondwani G. H. Katundu, Victoria Mukhula, Tamara Phiri, Chimota Phiri, Florence Filisa-Kaphamtengo, Pascal Chipewa, George Chirambo, Mwapatsa Mipando, Henry C. Mwandumba, Adamson S. Muula, Johnstone Kumwenda

**Affiliations:** 1 Department of Biomedical Sciences, Kamuzu University of Health Sciences, Blantyre, Malawi; 2 Malawi-Liverpool Wellcome Clinical Research Program, Kamuzu University of Health Sciences, Blantyre, Malawi; 3 Department of Medicine, Kamuzu University of Health Sciences, Blantyre, Malawi; 4 Department of Medicine, Queen Elizabeth Central Hospital, Blantyre, Malawi; 5 Kamuzu Central Hospital, Lilongwe, Malawi; 6 Blantyre to Blantyre Research Facility, Kamuzu University of Health Sciences, Blantyre, Malawi; 7 Department of Community and Environmental Health, Kamuzu University of Health Sciences, Blantyre, Malawi

**Keywords:** Dyslipidaemia, Diabetes mellitus, Hypertension, Queen Elizabeth Central Hospital, Malawi

## Abstract

**Background:**

Dyslipidaemia drives the process of atherosclerosis, and hence a significant modifiable risk factor complicating hypertension and diabetes. In Malawi, the prevalence, screening and management of dyslipidaemia among persons with diabetes mellitus have not been reported. This study aimed to investigate the prevalence, biochemical characteristics, screening and management practices for dyslipidaemia among persons with diabetes mellitus, hypertension, and diabetes mellitus and hypertension comorbidity at Queen Elizabeth Central hospital in Blantyre, Malawi.

**Methods:**

This was a cross-sectional study conducted in 2021. A total of 256 adult participants (diabetes mellitus = 100); hypertension = 100; both conditions = 56) were included. Medical data and anthropometric measurements were recorded. Blood samples were analysed for HbA1C and serum lipids. Associated risk factors for dyslipidaemia were also assessed.

**Results:**

Dyslipidaemia was prevalent in 58%, 55%, and 70% of participants with diabetes mellitus, hypertension, and both conditions. Low-density lipoprotein cholesterol (LDL-C) dyslipidaemia was the most common in all participant groups. Participants with both diabetes and hypertension had 2.4 times (95% CI 1.2–4.6) increased risk of LDL-C dyslipidaemia than those with diabetes alone (*p* < 0.02). Being overweight or obese and age over 30 years were risk factors for dyslipidaemia in participants with diabetes mellitus alone (OR 1.3 (95% CI 1.1–1.6), *p* < 0.04, and OR 2.2 (95% CI 1.2–4.7) (*p* < 0.01), respectively. Overweight and obesity predicted LDL-C dyslipidaemia in hypertensive patients (OR 3.5 (95% CI 1.2–9.9) *p* < 0.001). Poorly controlled hypertension and the use of beta-blockers and thiazide diuretics predicted dyslipidaemia among patients with both diabetes mellitus and hypertension (OR 6.50 CI 1.45–29.19; and OR 5.20 CI 1.16–23.36 respectively). None of the participants had a lipogram performed before the study or were on lipid-lowering therapy.

**Conclusions:**

Dyslipidaemia with LDL-C derangement was highly prevalent, especially in individuals with both diabetes mellitus and hypertension, and there was absent use of lipid-lowering therapy. Screening and managing dyslipidaemia should be reinforced to reduce the risk of cardiovascular complications in this population at increased risk.

## Background

Cardiovascular diseases (CVDs), mainly ischaemic heart disease and stroke, are the leading causes of morbidity and mortality globally and in Sub-Saharan Africa [1–3]. Dyslipidaemia, hypertension, and diabetes mellitus (DM) are major risk factors for CVDs [2, 4]. Dyslipidaemia drives the process of atherosclerosis [1] and is a significant modifiable risk factor among persons with hypertension and diabetes [5–7]. Dyslipidaemia, through its associated complications, accounts for over 2 million annual deaths and nearly 30 million disabilities globally [8].

Classically, diabetic dyslipidaemia presents with elevated triglycerides (TG), reduced high-density lipoprotein cholesterol (HDL-C) and normal to mildly elevated low-density lipoprotein cholesterol (LDL-C) associated with small dense low-density lipoprotein (sdLDL) particles [9, 10]. The pattern results from the overproduction of TG-rich very-low-density lipoprotein (VLDL) particles in the liver and increased exchange of TG in VLDL for cholesteryl esters in HDL and LDL-producing sdLDL [9–11]. A complex interaction of genetic and biochemical parameters in diabetic dyslipidemia has also been described [12]. Decreased serum adiponectin levels, a downregulation in the expression of genes expressing adiponectin such as rs2241766 and rs1501299 and their receptors such as ADIPOR1 and ADIPOR2, and increase levels of oxidative stress have been associated with diabetic dyslipidaemia [12, 13]. Furthermore, higher concentrations of biochemical factors such as plasma C-reactive protein and 4-hydroxynonenal adducts have identified as markers of diabetic dyslipidaemia [14]. On the other hand, the typical pattern of dyslipidaemia in hypertension includes increased total plasma cholesterol (TC), TG and LDL-C, decreased HDL-C and changes in LDL-C composition including sdLDL-C, and increased electronegativity of LDL-C [15, 16]. In both diabetic dyslipidaemia and dyslipidaemia in hypertension, elevated LDL-C influences atherosclerosis and causes CVD [17, 18]. The management of dyslipidaemia to reduce the risk of CVD, therefore, targets the reduction of LDL-C concentration [18].

Primary and secondary prevention of CVD is essential in patients with DM and hypertension. An assessment of cardiovascular risk, a discussion of a heart-healthy lifestyle and the relevance of lipid-lowering therapy should be discussed between the clinician and patient [19–21]. For patients with DM aged between 40 and 75 years without other known complications, moderate statin therapy is indicated, in addition to lifestyle modifications, to achieve an LDL-C concentration of < 70 mg/dl [18, 19]. DM complicated with other major risk factors for CVD, such as hypertension, warrants consideration of high-intensity statins to reduce the LDL-C by > 50% [18, 19]. On the other hand, patients with uncomplicated hypertension require screening for dyslipidaemia annually, and in the presence of dyslipidaemia, they should be managed with lifestyle modifications and lipid-lowering drugs to achieve LDL-C levels of < 100 mg/dl [4, 19, 22, 23].

A reduction of LDL-C levels by 38.7 mg/dL (1 mmol/L) reduces the 5-year incidence of CVD by 23% [24, 25], and optimal clinical benefits can be seen up to an LDL-C level of 40 mg/dl [26]. Despite the benefits of effective management, dyslipidaemia is often unscreened and undertreated across many regions of the world, including sub-Saharan Africa [27–30]. Malawi lacks data on the screening and management of dyslipidaemia among patients with DM and hypertension in many health facilities, including tertiary referral hospitals. However, the estimated prevalence of hypertension and DM in the general adult population is about 33% and 7%, respectively [31, 32]. This study investigated the pattern and prevalence of dyslipidaemia among patients with DM, hypertension, and comorbidity of DM and hypertension at a tertiary hospital in Blantyre, Malawi. The associated risk factors for dyslipidaemia, previous screening and management of the patients at the hospital were also investigated.

## Methods

### Study design and setting

The study was cross-sectional and was conducted at the DM and Hypertension clinics of the Queen Elizabeth Central Hospital (QECH) in Blantyre, Malawi. QECH is the largest tertiary referral hospital in the Southern Region of Malawi. Enrolment of study participants took place between March and July in 2021.

### Inclusion and exclusion criteria

The study population were adults aged 18 years and above with hypertension, type 1 and type 2 DM attending the DM and Hypertension clinics at QECH. We excluded pregnant participants, those with an incomplete medical history, participants with fever or history of an active infection, and those from whom blood sample collection was not successful.

### Study population and sampling strategy

Participants were enrolled when they attended the DM and Hypertension clinics over the study period. Due to the COVID-19 pandemic, not more than 150 patients were being seen per month at each clinic respectively. Consecutive sampling was used, and a total of 256 participants were recruited.

Sociodemographic data were collected using interviewer-guided questionnaires. Collected data included sociodemographic data such as age, sex, highest education level and employment status, medical diagnoses including HIV and the dates of diagnosis, current medication, exercise history, smoking and alcohol intake and history of any cardiovascular event. Temperature and blood pressure measurements were also recorded. Weight and height assessments were done, from which the body mass index (BMI) was calculated and classified according to the World Health Organization (WHO) classification [33]. Waist and hip circumferences were also measured. Data on whether the participants had their BMI measured and blood lipid levels ever tested within the past 12 months from the date of the study was also recorded. For participants with DM, the type of DM was recorded, and data on pharmacotherapy for DM and hypertension were documented.

A venepuncture was performed, and a blood sample was collected. Random blood glucose and glycated haemoglobin (HBA1c) were performed on whole blood samples using the Rossmax HS200 analyser (USA) and the HBA1c EZ 2.0 (Wuxi BioHermes Biomedical Technology Co., Ltd, China), respectively. The rest of the blood samples were collected in a vacutainer tube without an anticoagulant and stored on ice. Samples were then centrifuged within three hours and stored in -80-degree Celsius freezers until lipogram analysis. Samples were then analysed for LDL-C, TG, TC and HDL using an automated Erba XL640 (USA) by a qualified laboratory technologist.

### Definition of dyslipidaemia

Using the American Association of Clinical Endocrinologists and American College of Endocrinology guidelines for the management of dyslipidaemia and prevention of cardiovascular disease guidelines [4], dyslipidaemia was defined as any of the following abnormalities: TC ≥ 200 mg/dl, TG ≥ 150 mg/dl, LDL-C ≥ 100 mg/dl and HDL-C < 40 mg/dl. The presence of a single abnormal lipid parameter (TC, TG, HDL-C or LDL-C) was classified as isolated dyslipidaemia. The presence of two abnormal lipid parameters (elevated TG, low HDL-C or elevated LDL-C) was classified as combined dyslipidaemia. The abnormality of three indices, TG, HDL-C and LDL-C, was ranked as mixed dyslipidaemia.

### Statistical analysis

Data were entered in Microsoft Excel spreadsheets and statistical analysis was performed using Stata16 (StataCorp, USA) software. Descriptive statistics were expressed as the means or medians for continuous data such as age and lipid concentrations and proportions for categorical data. The prevalence of dyslipidaemia was calculated in the participants with DM, hypertension and among participants with both comorbidities. The Chi squared test or Fisher’s exact test for independent variables was used to compare categorical data. For hypothesis testing, the t-test was used to analyse the differences in mean difference in lipid concentration parameters between any two groups. Logistic regression analysis was used to compare the differences in risk of dyslipidaemia between participants of different age groups, socio-economic status, or BMI categories. Multivariate logistic regression analysis was used to account for confounding. Correlations between the variables were tested using Pearson’s Product or Spearman’s correlation test, depending on the data normality. In all cases, a *p *value < 0.05 was considered significant.

## Results

### Characteristics of the study participants

**Fig. 1:**
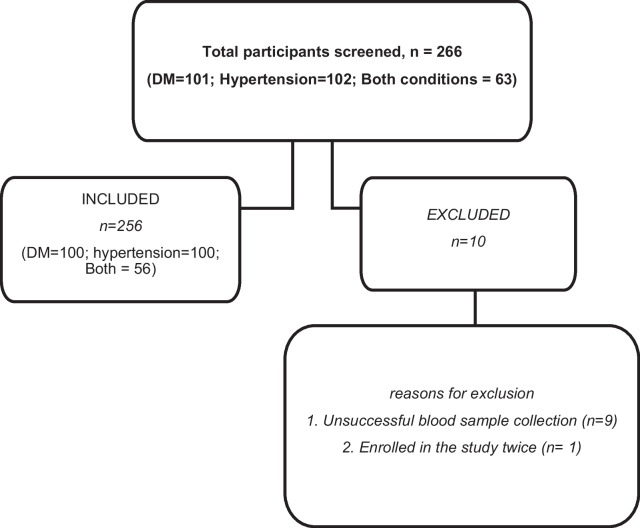
Strobe diagram illustrating the number of participants screened, those who were included and the reasons for exclusion in the study

Figure 1 summarises the number of participants screened for the study and the reasons for exclusion from the study. A total of 266 participants were screened and of these, 10 participants were excluded from the study either due to unsuccessful blood sample collection or having been enrolled in the study already.

Of the participants who had DM only, 84% had T2DM, while 16% had T1DM. Of the participants who had both DM and hypertension, 96% had T2DM, and only 4% had T1DM.

### Sociodemographic and clinical characteristics of the participants

Table 1 shows the sociodemographic and clinical characteristics of the participants. Notably, there were more females in each group (≥ 60%) than males. Most of the participants were within the middle-age group with a median age of 44 years (IQR 37–51 years), 52 years (IQR 42–62 years) and 57 years (IQR 51–57 years) for DM, hypertension, and DM with hypertension comorbidities, respectively. The prevalence of HIV ranged from 22 to 26% in the three groups. The median time from the date of diagnosis for the conditions was three years, five years and six years for DM, hypertension and DM with hypertension, respectively. Most participants in all the disease condition groups were either overweight or obese 55%, 83% and 84%, respectively, in the DM, hypertension and DM with hypertension groups. There was poor glycaemic control in the participants who had DM whereby 88% and 67% of the participants had HBA1c > 7% in the DM only and DM with hypertension groups. For participants with hypertension, there was poor blood pressure control with median systolic and diastolic pressures of 148 mmHg (IQR 136–170 mmHg) and 93 mmHg (IQR 83–103 mmHg) for hypertension only group and 152 mmHg (IQR 135 – 166 mmHg) and 92 mmHg (IQR 84-101 mmHg) for the DM with hypertension group.

**Table 1:** Sociodemographic and clinical characteristics of the participants

	DM (N = 100)	Hypertension (N = 100)	DM and hypertension (N = 56)
Characteristic			
Sex (female) (%)	63	60	73
Age			
Median (IQR)	44 (37–51)	52 (42–62)	57 (51–57)
HIV status			
Positive (%)	25	26	22
Negative (%)	70	68	68
Unknown (%)	5	6	10
Type 2 DM (%)	82	–	96
Duration of diagnosis			
Median years (IQR)	3 (1–6)	5 (1–11)	6 (3–12) HTN 7 (3–17) DM 6 (3–12)
BMI			
Median (IQR)	26 (23–31)	29(26–34)	29 (26–34)
Underweight (%)	5	0	0
Normal (%)	37	17	16
Overweight (%)	28	35	40
Obese (%)	30	48	44
High waist-hip ratio (%)	57	47	77
HBA1C			
Median (IQR)	11 (9–14)	–	8.3 (7–12)
> 7%	88%	–	67%
RBG			
Median mg/dl (IQR)	223 (153–281)	–	146 (107–210)
Blood pressure			
Median Systolic (IQR) mmHg	127 (116–139)	148 (136–170)	152 (135–166)
Median Diastolic (IQR)	83 (77–91)	93 (83–103)	92 (84–101)
Previous CVD (%)			
Stroke (%)	0	14	4
IHD (%)	0	8	0

Regarding medication for the DM-only group, 38% were on insulin, 52% were on oral anti-glycaemic medication (glibenclamide, metformin or a combination), and 10% were on metformin and insulin. The most frequent hypertensive medication for the hypertension-only group was hydrochlorothiazide (73%). Other antihypertensive medications were: amlodipine, enalapril, atenolol, nifedipine, propranolol furosemide and spironolactone. Participants with both DM and hypertension had a combination of pharmacotherapy for both conditions. None of the three group participants were on lipid-lowering therapy. There were 14% and 4% previously recorded cases of stroke in the hypertension-only group and the DM with hypertension group, respectively, and none in the DM group. For the history of ischaemic heart disease, 8% of participants in the hypertension group reported previous episodes, while none reported such episodes from the other disease groups.

### Prevalence and patterns of dyslipidaemia in the study population

**Fig. 2:**
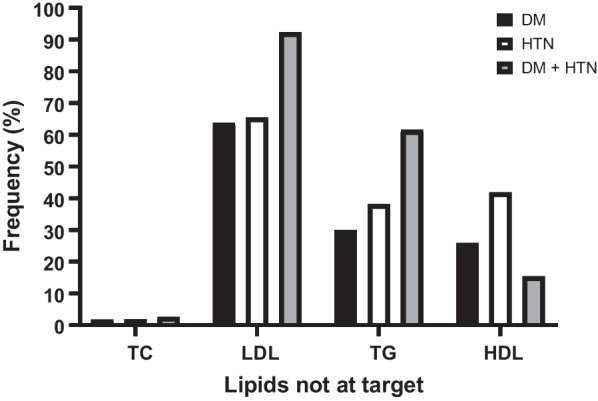
Individual lipid abnormalities among the three participant groups. HTN: hypertension

Dyslipidaemia was prevalent in 58% of the diabetic group, 55% of the hypertensive group and 71% of the participants with DM and hypertension comorbidities, respectively. Figure 2 shows the individual lipid abnormalities among the three participant groups. LDL-C elevated in 63.8% of the DM participants, 65.45% of participants and 92.3% of the participants with both comorbidities. TG was the second most frequent lipid abnormality, followed by HDL-C, and the least frequent lipid abnormality was TC.

Table 2 depicts dyslipidaemia prevalence and biochemical types among the three participant groups. LDL dyslipidaemia in isolation or in combination with other lipid abnormalities was the most prevalent lipid abnormality in all three participant groups. Combined dyslipidaemia was the second most frequent type among all the three study population groups, with the TG + LDL type being the most frequent combined type of dyslipidaemia.

**Table 2:** Dyslipidaemia patterns among the participant groups

Pattern of dyslipidaemia	DM *(n* = *100)*	Hypertension (n = 100)	DM + hypertension (n = 56)
	n (%)	n (%)	n (%)
Isolated	31 (53.5)	31 (52.7)	15 (38.5)
High TC	1 (1.7)	1 (1.8)	1 (2.6)
High LDL-C	16 (27.6)	14 (25.5)	13 (33.3)
High TG	6 (10.3)	0 (0)	1 (2.6)
Low HDL-C	8 (13.8)	14 (25.5)	0 (0)
Combined	23 (39.7)	20 (36.4)	20 (51.3)
High LDL + high TG	14 (24.1)	15 (27.3)	18 (46.2)
High LDL + low HDL	3 (5.2)	3 (5.5)	1 (2.6)
Low HDL + high TG	6 (10.3)	2 (3.6)	1 (2.6)
Mixed (high LDL + high TG + low HDL)	4 (6.9)	4 (7.3)	4 (10.3)

### Associated risk factors of dyslipidaemia in the DM group

Table 3 displays the association of selected variables with dyslipidaemia among participants with DM only. Notably, among these participants, dyslipidaemia was positively and statistically significantly associated with overweight and obesity, and age more and 30 years old OR 1.30 (95% CI 1.01–1.68) *p* = 0.04; and OR 2.15 (95% CI 1.1–4.72), p < 0.01. Age more than 30 years old emerged an independent predictor of DM dyslipidaemia upon multivariate analysis after accounting for BMI, DM type and waist to hip ratio. In Addition, HIV infection was associated with HDL dyslipidaemia (OR 2.356) (CI 1.11–5.01), *p* < 0.03.

**Table 3:** Association between selected variables and dyslipidaemia in participants with DM

Variable	Reference	Odds ratio	95% Conf. interval	*p*-value
Sex	Male	1.14	0.88–1.48	0.303
Overweight and obesity	Normal BMI	1.30	1.01–1.68	0.036
HBA1C ≥ 7%	< 7%	0.98	0.69–1.39	0.902
Age > 30 years?	No	2.15	1.10–4.72	0.003
HIV	Negative	0.92	0.68–1.25	0.590
Exercise	< 30 min/day	0.90	0.72–1.13	0.388
DM type	Type1	1.39	0.91–2.14	0.057
High waist-hip ratio	Normal	1.23	0.96–1.58	0.085

### Associated risk factors of dyslipidaemia in the hypertension group

Dyslipidaemia in the hypertension group was not statistically significantly associated with sex, age, overweight or obesity, HIV status, poorly controlled hypertension or waist-to-hip ratio (See Table 4). Nevertheless, overweight and obesity were associated risks for LDL dyslipidaemia (OR 3.49, CI 1.23–9.89) *p* < 0.001).

**Table 4:** Association between selected variables and dyslipidaemia in participants with hypertension

Variable	Reference	Odds ratio	95% Conf. interval	*p*-value
Sex	Male	0. 87	0.69–1.11	0.206
Overweight and obesity	Normal BMI	1.43	0.90–2.27	0.058
Poorly controlled HTN	controlled	1.03	0.78–1.36	0.843
Age > 30 years?	No	1.46	0.65–3.29	0.220
Use of thiazide diuretics and/or beta-blockers	No use	0.95	0.70–1.30	0.760
HIV	Negative	1.04	0.80–1.37	0.764
Exercise	< 30 min/day	1.08	0.83 1.40	0.555
High waist-hip ratio	Normal	1.21	0.95–1.54	0.123

### Associated risk factors of dyslipidaemia in participants with both DM and hypertension

**Fig. 3:**
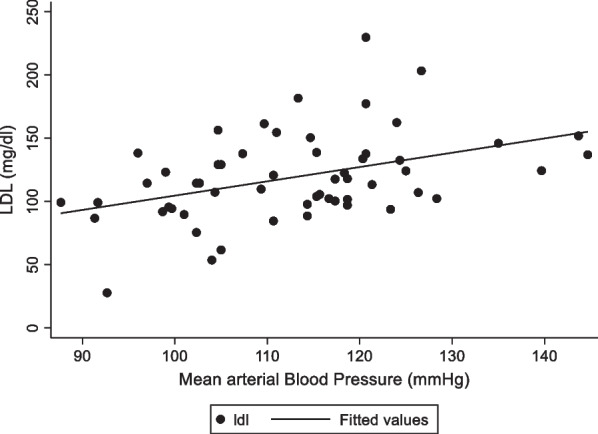
Correlation between LDL concentration and Mean arterial Blood Pressure. LDL concentration positively correlated with mean arterial blood pressure (Spearman’s rho = 0.429, *p* = 0.001)

In participants who had both DM and hypertension, poorly controlled hypertension and use of thiazide diuretics and/or beta-blockers were positively associated with dyslipidaemia (See Table 5). Moreover, as depicted in Fig. 3, LDL levels positively correlated with this group’s mean arterial blood pressure (Spearman’s rho = 0.429, *p* < 0.01). Upon multivariate analysis, both poorly controlled hypertension and use of thiazide diuretics and/or beta-blockers were independent predictors of dyslipidaemia (OR 6.50 CI 1.45–29.19; and OR 5.20 CI 1.16–23.36 respectively).

**Table 5:** Association between selected variables and dyslipidaemia in participants with both DM and hypertension

Variable	Reference	Odds ratio	95% Conf. interval	*p*-value
Sex	Male	0.76	0.60–0.96	0.076
Overweight and obesity	Normal BMI	1.30	0.67–1.35	0.791
HbA1C ≥ 7%	< 7%	1.13	0.80–1.59	0.468
Poorly controlled HTN?	No	1.75	1.06–3.00	0.003
Age > 30 years?	No	0.76	0.66–0.89	0.579
HIV	Negative	0.97	0.69–1.37	0.878
Exercise	< 30 min/day	0.84	0.64–1.11	0.290
DM type	Type1	0.76	0.65–0.88	0.428
Use of thiazide diuretics and/or beta-blockers	No use	1.55	1.10–2.29	0.011
High waist-hip ratio	Normal	1.32	0.840–2.08	0.137

## Discussion

In this study, we observed a high prevalence of dyslipidaemia among patients with DM, hypertension, and both DM and hypertension. LDL-C dyslipidaemia was the most common disorder in all the study groups. Being overweight or obese and age of more than 30 years were significantly associated with dyslipidaemia in the population with DM, while overweight and obesity were significant risks for LDL-C in participants with hypertension alone. In participants with DM and hypertension comorbidity, poorly controlled hypertension was associated with dyslipidaemia. None of the participants were not on any lipid-lowering therapy and had no previous lipid profiles performed at the clinics before this study.

Dyslipidaemia, particularly high LDL-C, causes approximately 4.3 million deaths worldwide and about 95 million Disability Adjusted Life-years (DALYs) globally [34]. Mortality owing to elevated LDL-C is increasing globally, mainly due to an exponential increase in low and middle-income countries, compared to high-income countries [35, 36]. The averted deaths by the high-income countries were achieved through efficient screening, promotion of healthier lifestyles, and aggressive pharmacotherapy and management of dyslipidaemia targeting LDL-C [35–37]. From the results of the present study, at least 50% of the study participants were at increased risk of cardiovascular events due to dyslipidaemia. A similar high prevalence of dyslipidaemia has been reported in other African countries among people living with DM and hypertension [38–40]. The lack of screening and management of dyslipidaemia in the present study highlights the gap in addressing the burden associated with dyslipidaemia in high-risk individuals with DM and hypertension in Malawi, hence contributing to the high burden associated with CVDs [34].

Poor screening and management of dyslipidaemia discovered in this study is arguably multifactorial. Screening for dyslipidaemia requires performing lipid profiles which are relatively costly to run, and the government hospital laboratories often have the reagents out of stock [41], and lipogram reagents are not prioritised as essential medical supplies. In addition, there are no local Malawian clinical practice guidelines which reinforce the implementation of routine screening for dyslipidaemia and its management. Furthermore, sub-optimal knowledge and experience about dyslipidaemia among the public and healthcare professionals in the face of heavy workload have been underscored as a significant contributor to poor screening and management of dyslipidaemia in Africa [42, 43]. In addition, inexperienced or junior clinical personnel may not consider performing a lipogram in people with DM or hypertension essential [42, 43].

Despite the known benefit of lipid-lowering medications when indicated in patients with DM and hypertension [44], pharmacotherapy for dyslipidaemia is often unavailable in many African public health facilities and unaffordable in private pharmacies [45, 46]. Indeed, in Malawi, as in other countries, efforts are required from governments, stakeholders, and manufacturers to ensure the availability and access to affordable lipid-lowering therapy [8]. In the case of the present study, pharmacotherapy would be targeted at LDL-C as it was the most common lipid abnormality. Clinical trials have shown that statins, ezetimibe and protein convertase subtilisin/kexin type 9 (PCSK9) inhibitors can reduce high LDL-C by at least 50% of the baseline [4, 24, 47, 48]. Practically, achieving the recommended LDL-C goals often demand the use of a combination of lipid-lowering therapy [48]. However, ideally, all patients with an elevated ASCVD risk should receive the maximum tolerated statin dose as the first-line treatment unless contraindicated [25, 48, 49]. Considering the high cost of lipid profiles for government health facilities in a setting such as Malawi and the high prevalence of dyslipidaemia in the study population, we need to consider whether blanket statin therapy in individuals with DM above the age of 30 years and those with DM and hypertension comorbidities would be indicated [18, 19].

At least 20% of the participants in the present study had HIV infection. Careful consideration of the appropriate lipid-lowering therapy, whether statins or fibrates, in patients with dyslipidaemia and on ART is essential [22]. Atorvastatin has been considered the statin of choice in patients with HIV [22]. However, simvastatin is contraindicated and rosuvastatin is not recommended due to the high risk of drug interactions with ARVs [50]. Before prescribing lipid-lowering therapy to patients on ART, clinicians are encouraged to refer to the information on the drug-drug interactions between lipid-lowering agents and ARVs [22] available from www.hiv-druginteractions.org [51].

We found high rates of overweight and obesity in the study participants, and abdominal obesity, marked by the high waist-to-hip ratio, was also highly prevalent. Being overweight and obese positively influences dyslipidaemia [11], as was also observed in this study. Positive lifestyle modifications such as physical activity, as recommended by the WHO and appropriate cardiovascular-friendly dietary habits should be reinforced as part of the significant elements of lifestyle therapy [49]. Malawi now has in-country-trained dieticians working in the tertiary level facilities who would assist with health education and lifestyle modification [52].

In this study, we noted poor glycaemic control in participants with DM and poor hypertension control for individuals with hypertension. Poor glycaemic management has been shown to contribute to dyslipidaemia [53] adversely and likely contributed to dyslipidaemia in the participants with DM in this study. Similarly, poor hypertension control correlates with LDL-C [54, 55], this was observed in this study, and it predicted dyslipidaemia in participants with both hypertension and DM. The reasons for poor glycaemic and hypertension control were beyond the scope of this study but are a matter of concern as they influence the risk for CVDs.

This study had some limitations. The study's cross-sectional design precluded any temporal association between the risk factors and dyslipidaemia. We did not investigate the influence of adiponectin in the pathophysiology of obesity and dyslipidaemia [12, 56] in the present study. Future stidies should elucidate the role of adiponectin in dyslipidaemia, including the potential therapeutic role in this population. In addition, this study was performed at one tertiary hospital in Malawi, and hence may not be generalised to other rural Malawian settings. More extensive studies which would include rural settings would be useful to inform country-wide clinical guidelines on screening and management of dyslipidaemia in these population groups of patients with DM and hypertension.

## Conclusions

Our study draws attention to the high prevalence of dyslipidaemia in persons with DM and hypertension, particularly in those with both these comorbidities and the absence of lipid-lowering therapy to reduce the risk of CVDs. It highlights the need for clinical guidelines to incorporate and reinforce screening and treatment of dyslipidaemia in managing the conditions. The development of such guidelines should consider the current challenges in public health service delivery. The use of statins for primary and secondary prevention of ASCVD should be stressed in patients with dyslipidaemia. The potential for a blanket treatment in patients at the highest risk such as those with DM and hypertension comorbidities, may also be considered. The study also highlights the need to address the high prevalence of overweight and obesity and poor glycaemic and hypertension control to reduce the risk of CVDs. More extensive studies in different settings of Malawi are necessary to provide generalised data which would inform the magnitude of dyslipidaemia in patients with DM and hypertension in Malawi. Additionally, Further studies on the availability and impact of statin use in public hospitals in Malawian patients with DM and hypertension are required.

## Data Availability

The datasets used and/or analysed during the current study are available from the corresponding author upon reasonable request.

## Abbreviations

ASCVDs: Atherosclerotic cardiovascular diseases
BMI: Body mass index
CI: Confidence interval
COVID-19CVD: Coronavirus disease 2019 Cardiovascular disease
COMREC: College of Medicine Research Ethics Committee
CVDs: Cardiovascular diseases
DALYS: Disability Adjusted Life-years
DM: Diabetes mellitus
HbA1c: Glycated haemoglobin
HDL-C: High-density lipoprotein cholesterol
HTN: Hypertension
IQR: Interquartile range
LDL-C: Low-density lipoprotein cholesterol
OR: Odds ratio QECH: Queen Elizabeth Central Hospital
RBG: Random blood glucose
SDI: Sociodemographic index
T1DM: Type 1 diabetes mellitus
T2DM: Type 2 diabetes mellitus
TG: Triglyceride
USA: United States of America
VLDL: Very low-density lipoprotein
WHO: World Health Organization
